# Health System Outcomes in BRICS Countries and Their Association With the Economic Context

**DOI:** 10.3389/fpubh.2020.00080

**Published:** 2020-03-31

**Authors:** Piotr Romaniuk, Angelika Poznańska, Katarzyna Brukało, Tomasz Holecki

**Affiliations:** ^1^Department of Health Policy, Faculty of Health Sciences in Bytom, Medical University of Silesia in Katowice, Bytom, Poland; ^2^Department of Health Economics and Management, Faculty of Health Sciences in Bytom, Medical University of Silesia in Katowice, Bytom, Poland

**Keywords:** BRICS, health system outcomes, health system performance, health system determinants, developing countries

## Abstract

The aim of the article is to compare health system outcomes in the BRICS countries, assess the trends of their changes in 2000−2017, and verify whether they are in any way correlated with the economic context. The indicators considered were: nominal and per capita current health expenditure, government health expenditure, gross domestic product (GDP) per capita, GDP growth, unemployment, inflation, and composition of GDP. The study covered five countries of the BRICS group over a period of 18 years. We decided to characterize countries covered with a dataset of selected indicators describing population health status, namely: life expectancy at birth, level of immunization, infant mortality rate, maternal mortality ratio, and tuberculosis case detection rate. We constructed a unified synthetic measure depicting the performance of individual health systems in terms of their outcomes with a single numerical value. Descriptive statistical analysis of quantitative traits consisted of the arithmetic mean (xsr), standard deviation (SD), and, where needed, the median. The normality of the distribution of variables was tested with the Shapiro–Wilk test. Spearman's rho and Kendall tau rank coefficients were used for correlation analysis between measures. The correlation analyses have been supplemented with factor analysis. We found that the best results in terms of health care system performance were recorded in Russia, China, and Brazil. India and South Africa are noticeably worse. However, the entire group performs visibly worse than the developed countries. The health system outcomes appeared to correlate on a statistically significant scale with health expenditures per capita, governments involvement in health expenditures, GDP per capita, and industry share in GDP; however, these correlations are relatively weak, with the highest strength in the case of government's involvement in health expenditures and GDP per capita. Due to weak correlation with economic background, other factors may play a role in determining health system outcomes in BRICS countries. More research should be recommended to find them and determine to what extent and how exactly they affect health system outcomes.

## Introduction

BRICS is a term used to describe a group of countries once considered to have similar characteristics of emerging economies. The term has been created from the first letters of their names, namely: B—Brazil, R—Russia, I—India, C—China, S—South Africa. Together, these countries account for 40% of the world's population and 25% of the world's gross domestic product (GDP). The first use of this term took place in 2001 in a paper published by J. O'Neill, not yet including the Republic of South Africa. After several years, the term BRIC has become widely used in financial markets, shaping the way investors, financiers, and decision makers view emerging markets (1).

The discussed group initially consisted of four countries: Brazil, Russia, India, and China. South Africa began to connect with the group since 2011. In 2006, however, the countries began closer political and economic cooperation. Despite this fact, today they still do not form a formal political alliance, primarily conducting activity whose main goal is to reform the United Nations, especially regarding the composition of the Security Council, increase the role of developing countries in the international arena, and create a new monetary system that will accelerate the pace of economic growth in these countries (2).

BRICS countries have been distinguished as one group mainly due to similarities in the level of economic development and the process of economic transformation. However, they also prioritize cooperation in the field of health. Many decision makers perceive BRICS as an instrument for changing global health (3, 4), although this does not necessarily translate into the uniformity of the characteristics of their health systems (5). Despite the fact that each country applies a different spectrum of detailed solutions and struggles with different challenges, all of them follow a scheme of mixed public and private responsibility and financing and all of them face similar issues related to underfinancing, staff shortages, and the limited capacity of a publicly financed system.

The Brazilian healthcare system provides all citizens with access to free and universal health protection, as stated in the 1988 Federal Constitution. Services are being provided at three levels: federal, state, and municipal; so is the scheme of system's financing (6). Primary care is one of the main pillars of the system here and remains the basic way to ensure greater access to health services for all citizens (6). Russian health care system, in turn, arises of the former Semashko model applied during communism era, having undergone a number of reforms since its end. Currently, it is based not only on mixed sources of revenues, namely, compulsory health insurance, which provides citizens with equal opportunities in access to health care, and state and regional budgets, but also on funds derived from various enterprises and institutions. In addition to the compulsory (public) health insurance system, there is an additional voluntary health insurance system in Russia. It operates on a commercial basis, at insurance rates. The state is the founder and owner of 68% of medical infrastructure. India, because of its diversity, has a mixed healthcare system, with the presence of public and private entities. Social insurance is compulsory and is applicable mainly in the formal sector (7, 8). As part of health insurance, there is the Central Government Health Scheme (CGHS)—and the Employees' State Insurance Scheme (ESIS). However, the most common source of spending in India are out-of-pocket payments, which makes India one of the most privatized healthcare sectors in the world (9). In China, the healthcare market is fueled by changes in the population structure and a government initiative seeking to ensure wider access to medical care (10). The Chinese health care system is shifting toward the privatization of health care, which in the future may be burdensome, especially for people living in rural areas (11, 12). The current problems of the Chinese system include the limits set by individual hospitals for the maximum cost of treatment for each patient and the duration of his/her stay in the hospital. Patients are treated in overcrowded hospitals; administrative systems do not have orderly structures. The lack of qualified employees is also a major obstacle (11, 12). In the Republic of South Africa, the healthcare system consists of two subsystems. The first of them is mostly funded by the public sector; the second is a rapidly growing private sector. The basic health care offered free of charge by the state is highly underfunded and overexploited (13). The challenges here are also the shortage of educated medical personnel and a pandemic of HIV infection, tuberculosis, cholera, and malaria. The occurrence of so many diseases correlates with a high burden on the national budget and rising costs of treatment (13, 14).

The context-defining basic problems for health systems in each of the BRICS countries differ from each other, but, in a general manner, they seem to be similar, being primarily determined by economic transitions and demographic phenomena. A question, however, appears, whether it translates into similar performance outcome in each of the five health systems, as well as whether this dependence between health system performance and economic context is indeed that clear, as suggested by general overview and the conviction that the general economic context does matter when analyzing the features and performance of health systems, where both the quantity and quality of health services delivered are determined by national economic potential, at least the size of GDP allocated to health care (15, 16). These issues constitute the basic subject of this paper.

## Aim

The principal aim of our study was to compare the health system outcomes in BRICS countries and assess the trends of their change in the period 2000–2017. We also aimed to verify whether the health system outcomes in this group of countries are in any way correlated with economic background, as defined by a set of measures referred to economic performance and health system financing.

## Methods

Our study covered a group of five countries commonly referred to as BRICS in a period of 18 years (2000–2017). The starting point of the analysis is determined by the similarity of circumstances accompanying the functioning of the economies of the countries being the subject of investigation, which also coincides with the time when they began to be treated as to some extent a homogenous group (2001), as described in the *Introduction* section. The last year covered is the last year with a full set of data available. In the study, we decided to characterize countries covered with a dataset of selected indicators describing population health status, namely: life expectancy at birth, level of immunization (BCG, DPT, HepB3, measles, Pol3), infant mortality rate, maternal mortality ratio (modeled estimate), and tuberculosis case detection rate. The basic keys for selection of the indicators were availability of data for the period covered by the study, as well as an assumption that they are the best available direct markers of health system outcomes [see i.e., (17, 18)].

Aiming to perform a comparative analysis of health system in BRICS countries, we constructed a unified synthetic measure depicting the performance of individual health systems in terms of their outcomes with a single quantitative measure. The foundation of our concept for the measure was similar to the one lying behind the Index of the Economic Aspects of Welfare, which, from the set of information on the consumption of goods, tries to deduce the level of affluence of a given social group, by surveying, e.g., the level and proportions of expenditure for consumption and investment purposes, value of free time, value of work in the household, or expenditure on health care in various calculation periods, or Human Development Index and Human Poverty Index assessing the situation of the population based on GDP per capita, life expectancy, or the level of education of citizens (19, 20). To calculate our health system outcome measure, we used a modified methodology previously applied for a similar study in countries of Central and Eastern Europe (21, 22). Like in the previous case, we used multidimensional comparative analysis. An algorithm including the unification and standardization of variables has been applied, followed by aggregation of the individual variables to construct one unified measure. Zeroed unitarization was used to normalize and unify the individual variables, accompanied by a transformation of destimulants into the stimuli, which enabled adoption of the set of diagnostic variables for the use as partial criteria in the process of assessing a complex phenomenon. (23–25). The zeroed unitarization method is based on the adoption of a fixed reference point, which is the range of the normalized variable. As a result of the normalization process, this variable takes a value in the range <0,1> in such a way that the transformed values reflect the assessment of the original elements of the variable matrix: the better the value of the element of the original matrix (x), the closer to 1 is the value of the corresponding element of the transformed matrix of normalized variables (Z). The normalization process is based on the following formulas (24, 26, 27):

$$\begin{array}{l} Z_{ij}=\frac{\underset{i}{\max}x_{ij}-x_{ij}}{\underset{i}{\max}x_{ij}-\underset{i}{\min}x_{ij}}(i=1,2,\ldots ,r;j=1,2,\ldots ,s)\\ Z_{ij}=\frac{x_{ij}-{\min x}_{ij}}{\underset{i}{\max}x_{ij}-\underset{i}{\min}x_{ij}}\;(i=1,2,\ldots ,r;j=1,2,\ldots ,s) \end{array}$$

where the first of these is used to normalize destimulant variables, while the second is used for primary variables being stimuli. The use of the above formulas enables the construction of a matrix of normalized variables, which, in turn, gives the possibility of ordering the subjects and creating rankings, as well as—as was the case with the algorithm we used for the purposes of our study—aggregation and subsequent creation of rankings.

The basic modification compared to study in CEE countries was a set of variables to be included in the synthetic measure, limited to only health status-related indicators, as mentioned above. We also decided to resign from weighting the components of synthetic measure, which has been performed in the original study, to avoid arbitrariness in determining the attributes, and assuming that all components are direct derivates of health system performance. Since individual cases of missing data appeared in time series covered by the analysis, we supplemented them by trend extrapolation. The missing primary data were relatively few and did not affect the final result of the study. The final score in terms of synthetic measure for each country has been obtained by calculating the mean value for all partial results acquired by a given country for each of the measures included as components of the synthetic measure.

We also decided to investigate whether the values of synthetic measure of health system outcomes in BRICS countries correlate with indicators defining the condition and features of their economies, as well as financial background for their health systems. The indicators considered were: current health expenditure (% of GDP), current health expenditure per capita (current US$), current health expenditure per capita, PPP (purchasing power parity; current international $), domestic general government health expenditure (% of current health expenditure), domestic general government health expenditure (% of GDP), domestic general government health expenditure (% of general government expenditure), GDP per capita, PPP (purchasing power parity; current international $), GDP growth (annual %), unemployment (% of total labor force; modeled ILO estimate), inflation (annual %), services share in GDP (%), industry share in GDP (%), agriculture, forestry, and fishing share in GDP (%). These are the indicators to depict the macroeconomic context for health system operation based on relatively easily identifiable measures, not only determining the general level of resources to be transferred to the health care system but also potentially having influence on degree of resignation from treatment for financial reasons, resignations from the purchase of drugs, degree of use of nonpublic healthcare, refusal to provide services (limits, queues, etc.), or individual funding of hospital stays (additional tests, medicines, meals, etc.), which subsequently have an impact on health system outcomes (28).

Descriptive statistical analysis of quantitative traits consisted of the xsr, SD, and where needed, the median. The normality of the distribution of variables was tested with the Shapiro–Wilk test. Since we observed nonnormal distribution of variables, Spearman's rho and Kendall tau rank coefficients were used for correlation analysis between measures. The significance level was set at *p* ≤ 0.05. For better reliability of obtained results, the correlation analyses had been supplemented with factor analysis.

All the data used for constructing synthetic measure and for correlation analyses were retrieved from the World Bank Database^1^. All statistical analyses were performed using MS Excel and Statistica v. 13.3 software. Since the study did not involve human participants, no ethical consent was required.

## Results

Table 1 presents descriptive statistics for synthetic outcome measure, its components, and a set of economic indicators included in the analysis. For the full set of original data for BRICS countries, please see Appendix 1.

**Table 1:** Descriptive statistics of the synthetic health system outcome measure and analyzed factors for BRICS countries in years 2000–2017.

	**Mean**	***SE***	**Median**	**Q1**	**Q3**	**Variance**	***SD***	**Min**	**Max**
Synthetic outcome measure	0.618	0.037	0.83	0.272	0.927	0.121	0.348	0.024	0.967
Immunization, BCG (%)	90.349	0.961	92.625	84.946	99	83.124	9.117	59	99
Immunization, DPT (%)	86.811	1.366	93	76.25	98	167.93	12.959	58	99
Immunization, HepB3 (%)	77.143	2.937	88.5	71	97	776.511	27.866	6	99
Immunization, measles (%)	87.589	1.475	96.5	82.25	98.75	195.705	13.989	56	99
Immunization, Pol3 (%)	85.871	1.482	93.375	71.06	98	197.539	14.055	57	99
Life expectancy	67.712	0.721	68.744	64.255	73.43	46.85	6.845	52.567	76.41
Tuberculosis detectection rate	73.833	2.274	87	59	87	465.421	21.574	33	100
Infant mortality rate	27.638	1.79	22.9	13.675	40.075	288.27	16.979	6.5	66.7
Maternal mortality rate	101.204	9.08	62.5	42.25	144.538	7419.393	86.136	14.915	374
health expenditure (% GDP)	5.906	0.229	5.038	4.244	7.486	4.723	2.173	3.246	11.772
Current health expenditure per capita (current US$)	371.392	36.135	287.611	66.969	524.049	117518.172	342.809	18.564	1407.708
Current health expenditure per capita, PPP (current international $)	701.042	51.176	626.503	226.767	1051.231	235712.8	485.503	82.294	1913.2
Government health expenditure (% of current health expenditure)	42.509	1.561	42.56	28.499	57.025	219.263	14.808	17.982	66.884
Government health expenditure (% of GDP)	2.547	0.123	2.936	1.305	3.422	1.358	1.165	0.712	4.703
Government health expenditure (% of general government expenditure)	8.226	0.328	9.112	6.287	9.761	9.655	3.107	2.745	14.06
GDP per capita, PPP (current international $)	10932.93	644.836	10408.024	6259.126	14105.02	37423185.47	6117.449	2156.69	26240.275
GDP growth (annual %)	5.031	0.394	5.15	2.963	7.86	13.967	3.737	−7.8	14.231
Unemployment rate (modeled ILO estimate)	10.013	0.954	6.603	4.4	9.823	81.995	9.055	2.268	33.473
Inflation (annual %)	6.374	0.446	5.75	3.786	8.418	17.898	4.231	−0.732	21.477
Services (% of GDP)	52.422	0.732	53.761	45.568	58.665	48.184	6.941	39.786	63.199
Industry (% of GDP)	30.683	0.829	28.855	26.456	31.582	61.877	7.866	18.353	47.559
Agriculture (% of GDP)	7.917	0.606	4.847	3.427	12.172	33.014	5.746	2.081	21.621

*BCG, Bacillus Calmette–Guérin vaccine; DPT, diphtheria, pertussis and tetanus vaccine; HepB3, Hepatitis B 3rd dose vaccination; Pol3, Polio vaccive; GDP, Gross Domestic Product; US$, United States Dollar; PPP, Purchasing Power Parity; ILO, International Labor Organization*.

The presented data shows that for the health system performance markers, the variance of values is relatively high. In most cases, we can observe a significant difference between mean value and median, which may be caused by the difference between values for one country in the group and the rest of them. The exception is life expectancy, where mean and median values are relatively close. We should note at the same time that these values are relatively low, although the difference between the minimum and maximum values is noteworthy, which may result both from differences between countries and the changes in observed value throughout the entire examined period. Apart from that, we should also pay attention to relatively low level of immunization (highest mean value for BCG), high infant and maternal mortality rates (with extremely high differences between minimum and maximum values), and low and highly diverse levels of tuberculosis detection rate. These features of health systems performance in BRICS countries are reflected in the values of the synthetic measure, which turn out to be highly diverse, scoring almost full scale from 0 to 1.

When it comes to the economic profile of the investigated group of countries, we can observe high diversity as well, despite the fact that the group used to be presented as having similar features and development routes. The share of services, industry, and agriculture in GDP vary from about 40 to 63%, 18 to 47%, and 2 to 22%, respectively. Although the diversity is high, we can definitely say that all of the countries differ from highly developed economies with domination of services in GDP. Also, the economic performance turns out to be diverse between countries and years, as it is apparent from the data on unemployment, inflation, GDP growth, and total values. Finally, the economic and financial background for the health system is also highly varying, as the presented data reveals. What, however, seems to be the common feature is low level of government expenditure on health care, even though in the group it remains highly diversified, varying from 18 to 67% of the total health expenditure.

**Figure 1:**
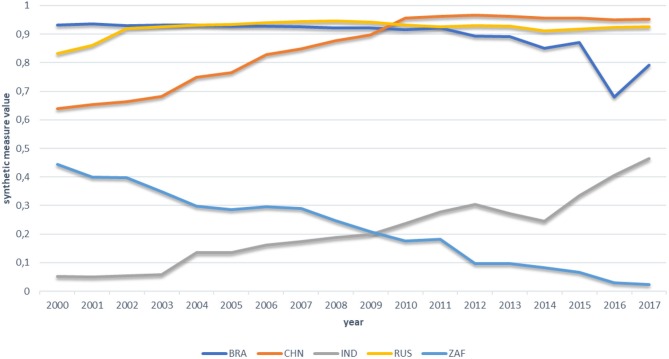
Synthetic measure of health system performance for BRICS countries in period 2000–2017. BRA, Brasil; CHN, China; IND, India; RUS, Russia; ZAF, South Africa.

To reveal differences in health system performance between examined countries, as well as to find out how the performance compared to each other changed over time, we calculated the synthetic measure of health system performance for each of the BRICS countries. Results are presented on Figure 1. A full matrix of the normalized variables included in the synthetic measure is presented in Appendix 2. The measure values do not provide any information in themselves. Their interpretation is only possible in comparative aspect, when they are analyzed within the investigated group.

Figure 1 reveals a few interesting facts. First, based on the measured values, we can divide the BRICS group into two subgroups, differing significantly in terms of health system performance. The first subgroup consists of Brazil, China, and Russia, with relatively similar results throughout the entire period. The second subgroup consists of India and South Africa, of which both notes visibly worse results than countries qualified to the first subgroup. The second interesting observation refers to the measured value changes over time. In the first subgroup, Russia gets a similar stable result since 2002. The result for Brazil also turns out to be highly stable, up to 2011, when a decline started to be visible, particularly strong between 2015 and 2016. Although in the next year we can observe the upward trend rebound, still at the end of examined period, Brazil got the worst result in this subgroup. Finally, in the case of China, we can observe constant and definite improvement in the subperiod 2000-2010. In the last year of this subperiod, China noted the best result of the entire BRICS group, and remains at the top of the ranking in all the following years.

In the second subgroup, the study also reveals important processes. First, in the case of India, although the result for this country remains visibly lower that for the three leading countries, we can observe significant and regular improvement starting in 2003. As a result, at the end of the examined period, the country managed to reduce the gap to leading countries of more than 40%. In the case of South Africa, in turn, we can observe the opposite trend. The country notes constant decline in terms of health system performance throughout the entire examined period, showing extremely low results at its end, which may even be a symptom of deep crisis in the South African health system.

Despite the fact of being perceived as a somewhat uniform group of countries, in terms of health system performance, as well as their economies, we have observed significant differences between them. We decided to apply Spearman's rho and Kendall tau rank coefficients to find whether health system performance and economic indicators remain in any kind of connection in the case of this group of countries. Results are presented in Tables 2, 3.

**Table 2:** Correlation between health system performance and economic factors.

	**Synthetic outcome measure**	**Immunization, BCG**	**Immunization, DPT**	**Immunization, HepB3**	**Immunization, measles**	**Immunization, Pol3**	**Life expectancy**	**Tuber detectection rate**	**Infant mortality rate**	**Maternal mortality rate**
Health expenditure (% GDP)	0.146084	0.236910	0.233878	0.291096	0.088360	0.254719	0.014644	0.391667	−0.189656	−0.152392
Health expenditure per capita	0.367329	0.364754	0.426269	0.592623	0.353114	0.416549	0.246113	0.639193	−0.571347	−0.475442
Health expenditure per capita, PPP	0.363477	0.373569	0.425788	0.569320	0.339018	0.419380	0.224777	0.667149	−0.574969	−0.473359
Government health expenditure (% of health expenditure)	0.547203	0.324978	0.494804	0.608219	0.414347	0.486649	0.093073	0.795639	−0.722282	−0.671002
Government health expenditure (% of GDP)	0.180129	0.192336	0.292778	0.390225	0.150267	0.293795	0.052212	0.541939	−0.391215	−0.271493
Government health expenditure (% of government expenditure)	0.070816	−0.007709	0.088910	0.243951	−0.023744	0.097218	−0.181472	0.376268	−0.220995	−0.209720
GDP per capita, PPP	0.516088	0.444090	0.521636	0.698963	0.474598	0.507949	0.292341	0.759777	−0.732563	−0.637572
GDP growth	0.098490	0.020566	0.072834	−0.064454	0.113838	0.044322	0.198749	−0.242524	0.066059	−0.017172
Unemployment	0.027895	−0.023444	0.031686	0.116562	−0.149989	0.062095	−0.343373	0.307822	−0.005755	−0.064809
Inflation	0.082570	0.166905	0.058163	−0.042579	0.124680	0.078896	−0.260633	0.382164	−0.108896	0.014678
Services (% of GDP)	−0.064263	−0.002187	−0.000389	0.144785	−0.096556	0.020862	−0.254723	0.281823	−0.038433	0.020909
Industry (% of GDP)	0.212347	−0.011992	0.058958	0.023534	0.144417	0.040499	0.181216	−0.016011	−0.215619	−0.353344
Agriculture (% of GDP)	−0.070321	0.049606	−0.054361	−0.252041	0.046622	−0.074709	0.307898	−0.420259	0.213791	0.229509

*Results for Spearman's rho coefficient test (statistically significant results in red)*.

**Table 3:** Correlation between health system performance and economic factors.

**variable**	**Synthetic outcome measure**	**Immunization, BCG**	**Immunization, DPT**	**Immunization, HepB3**	**Immunization, measles**	**Immunization, Pol3**	**Life expectancy**	**Tuber detectection rate**	**Infant mortality rate**	**Maternal mortality rate**
health expenditure (% GDP)	0.044694	0.159762	0.128433	0.158069	0.054192	0.162839	0.037703	0.260278	−0.168624	−0.099562
health expenditure per capita	0.264919	0.296029	0.317320	0.422113	0.257279	0.311015	0.209988	0.494820	−0.447914	−0.352221
health expenditure per capita, PPP	0.228464	0.294463	0.309017	0.400152	0.242021	0.301754	0.210487	0.518698	−0.460904	−0.355223
government health expenditure (% of health expenditure)	0.397253	0.233899	0.351049	0.447649	0.293582	0.344972	0.113608	0.626948	−0.546340	−0.473297
government health expenditure (% of GDP)	0.125593	0.196830	0.217168	0.269918	0.102070	0.223036	0.105618	0.373304	−0.291032	−0.187618
government health expenditure (% of government expenditure)	0.059933	0.043601	0.066430	0.179797	0.027099	0.096481	−0.045948	0.252084	−0.190881	−0.108832
GDP per capita, PPP	0.358801	0.315869	0.368174	0.507915	0.327255	0.350632	0.222971	0.616336	−0.583812	−0.483303
GDP growth	0.091635	0.027671	0.060973	−0.035495	0.079972	0.038330	0.127591	−0.174845	0.049213	−0.004003
Unemployment	0.020291	0.045832	0.023167	0.018958	−0.109265	0.078459	−0.233218	0.140545	0.052882	0.031121
Inflation	0.061673	0.106508	0.050595	−0.017109	0.097861	0.059939	−0.179026	0.274074	−0.077692	0.008005
Services (% of GDP)	−0.088140	0.034980	0.013751	0.055924	−0.039986	0.028555	−0.102122	0.179621	−0.058206	0.018512
Industry (% of GDP)	0.176529	0.038635	0.064606	0.005873	0.123115	0.050164	0.103620	−0.068187	−0.051212	−0.142089
Agriculture (% of GDP)	−0.069164	0.019840	−0.050076	−0.169305	−0.019993	−0.074860	0.152559	−0.327138	0.229578	0.151595

*Results for Kendall tau rank test (statistically significant results in red)*.

Despite some slight differences, both tests returned similar results. In the case of the synthetic measure, we can observe a statistically significant correlation with health expenditures per capita, government's involvement in health expenditures, GDP per capita, and industry share in GDP. All these correlations are positive, but, surprisingly, relatively weak, with the highest strength in the case of government's involvement in health expenditures and GDP per capita. However, in both cases, we can consider this strength as moderate at best.

In the case of individual components of the synthetic measure, we can observe that all those related to immunization tend to correlate at a statistically significant level primarily with indicators related to health expenditures. While this should not be considered as surprising, still the relatively low strength of this correlation must be noted, being strongest in the case of hepatitis. We observed the same situation in case of infant and maternal mortality rates. In both cases, the correlation is negative (the better results in terms of financial indicators, the lower the mortality is); interestingly, however, the strongest correlation in both cases appears to be not with those indicators that are related to health expenditures, but rather in the case of general level of economic development illustrated by GDP per capita. Spearman's test reveals a higher strength of correlation than Kendall's in these cases, reaching values that might be considered as a high level of correlation. In both cases, it is visibly higher than in the case of the synthetic measure. In the case of life expectancy, we observed a shift toward general economic indicators, with statistically significant, although still relatively weak, correlation with all of them, with the exception of GDP growth and industry share in GDP (also services share in GDP in the case of Kendall's test). Finally, only the tuberculosis detection rate turns to correlate with almost all the economic measures included in the analysis, with the only one exception for industry share in GDP. The correlation appears to be particularly strong in the case of government's engagement in financing health care, and the value of GDP per capita.

An observation that we found surprising is an almost complete lack of correlation between the health system outcome markers and the profile of economies, as illustrated by share of industry, services, and agriculture in GDP. In some cases, this is even more surprising, like when it comes to correlation between values of synthetic measures and the share of services in GDP, which, although extremely weak, is negative at the same time. Having regard to the fact that higher share of services should be connected with higher productivity of the economy, and—in result—better financial background for the health system, this observation is completely against this way of thinking.

**Figure 2:**
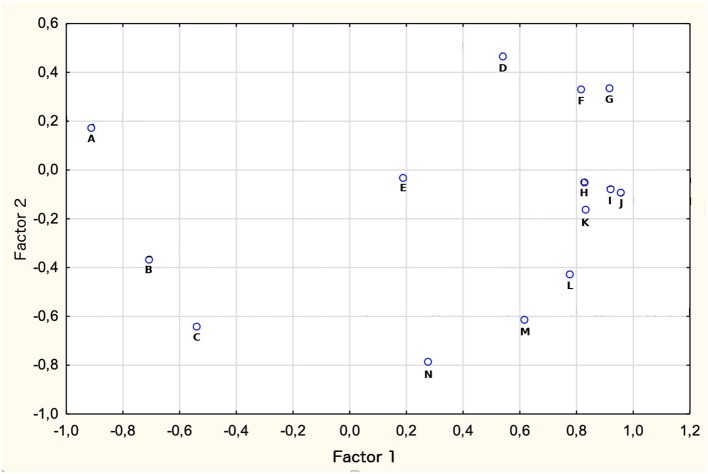
Correlation between health system performance and economic factors. Results for Factor analysis. A, Agriculture share in GDP (%); B, GDP growth; C, Industry share in GDP (%); D, Unemployment; E, Inflation rate; F, Health expenditures (% of GDP); G, Services share in GDP (%); H, Health expenditures per capita; I, Health Expenditures per capita (Purchasing Power Parity); J, Government expenditures on health (% of GDP); K, Government expenditures on health (% of total government expenditures); L, GDP per capita; M, government expenditure (% of total health expenditure); N, synthetic measure of health system outcomes.

The correlation test has been supplemented with factor analysis. The results are presented in Figure 2.

We found results of the factor analysis to be in line with previous results, confirming the low level of correlation between the synthetic measure of health system performance and economic indicators describing economies and financial background for the health systems in BRICS countries. Distribution of analyzed variables seems to be highly diffused between indicators, with just one aggregation of those related to features of health expenditures in examined countries, which may indicate their interconnectedness. The synthetic measure of health systems performance appears to be unaccompanied by any of the remaining variables, which might be interpreted as a relatively high level of its independence in relation to all the other variables.

## Discussion

There is only a limited number of studies concentrating on comparison of health systems in BRICS countries, or they concentrate on selected aspects of their performance, or the epidemiological status of countries. Petrie and Ki Tang (29) performed a similar study comparing BRICS countries' health system performance based on avoidable years of life lost indicator. Although based on different factors, the study showed a similar result to the one we observed in this study, with stable result in terms of health system performance for Russia, significant decline for South Africa, spectacular improvement in China, which managed to overtake Russia despite having a much lower level of economic indicators, and noticeable improvement in India, which overtook South Africa and gained distance to the leaders of the rank. Improvement in health system outcomes in China, although with strong variation between regions, has been also found in the study by Shi et al. (30). Petrie and Ki Tang (29) also noticed a different epidemiological profile in each of the countries, with other factors contributing in each of them to the premature mortality. Communicable diseases, with HIV/AIDS being at the first place, have been identified as the main factor responsible for epidemiological crisis here.

Mújica et al. (1) found some similar trends to those characterizing health system outcomes as illustrated in our study. One of them is decreasing life expectancy in South Africa appearing alongside with the rising burden of communicable diseases, which may then explain the drop we observed in result for this country and be the key health issue there. At the same time, South African health systems seem to not have delivered an adequate answer to this problem. The authors of the study at the same time mark the problem of high and rising social inequalities in the BRICS countries, which may contribute to poor health outcomes there. Pearce et al. (31) analyzed the productivity losses due to premature mortality from cancer in the five BRICS countries. The study findings are to some extent in line with the findings of our study, showing the highest level of economic loss caused by cancer-related deaths in relation to country GDP in South Africa followed by India. The best result has been identified in Brazil followed by Russia and China, although the differences between the first two are relatively low, while the result for China was only slightly better than the one for India, and slightly below the average for the entire group. The authors of the study also identified mortalities in BRICS countries as being more strongly connected to deficiencies in prevention, early detection, and treatment, when compared to developed countries (31). Rabiee et al. (32), in turn, analyzed the correlation between alcohol consumption and disease burden in BRICS countries. Trends and observation in this case are to some extent different from those arising of our study. The burden tends to be highest in Russia, however, with significant reduction starting from 2005 and at a similar and relatively stable level in the remaining countries. South Africa appeared to be an exception here, with a higher burden than in China, India, and Brazil and an observable rising trend up to 2000, and later decrease, which appeared to be against the trend in alcohol consumption (32).

Jakovljevic et al. (33) analyzed the trend in health expenditures in BRICS countries, finding constant increase in expenditure in all the five countries in the last two decades, with particularly clearly visible rising trends in the most recent 10 years. The trend was similar for government and private spending. Russia and Brazil were identified as leaders in terms of general health expenditures, and India appears to be at the bottom of the rank. This might explain the good result in terms of health system outcomes in Russia and Brazil, as found in our study, where also India, for the first half of period being subject of investigation, showed the weakest result. Nonetheless, the trends in health system expenditure do not explain the clear and constant drop of the result for South Africa and the improvement we observed in China, as they are not reflected in similar trends in expenditure. What is even more surprising, South Africa is generally characterized by a high level of both general and government spending, when compared to the other countries of the group. Also, the quality of services and patient satisfaction remain relatively high here (34). Hence, the study confirms our observation about the relatively weak correlation between health expenditures and health system outcomes, which then appears to be determined by other factors. Whether distribution of funds is among the determinants, having a crucial role should be subject to additional studies, although some evidence from China (12, 35, 36) shows that this factor is important for improving overall health system performance. Other policy features and the way they are developed and implemented may also play a role, as suggested by Gomez and Ruger (37). Kickbusch (38), in turn, suggested that the specific pattern of economic correlation with health in BRICS countries is determined by lifestyle and diet changes driven by growing wealth, which also correlates with high social inequalities and high level of air pollution. Lifestyle factors have also been paid attention in the study by Shukla et al. (39), identifying high prevalence of obesity in South Africa and high tobacco consumption in India as potential factors determining selected morbidities there. High alcohol consumption in Russia, a well-known fact, has also been raised. This turns the discussion again to the issue of fund flows and distribution, suggesting that programs aimed at control of modifiable individual behavior risk factors should be of particular focus for health systems in BRICS countries, where in general, a growing burden of noncommunicable diseases appears to be a rising trend (40). In Brazil, additionally, organizational issues appear to be of importance, with particular focus on human resources deficiencies, which negatively stimulate accessibility of services (6), while India need to increase health services coverage in lower socioeconomic groups (8).

The assessment of performance of health systems as presented in this study has any value in terms of comparative analysis. However, having regard to the fact, that Russia appears to be at the top of the ranking among BRICS countries in terms of health system outcome, and at the same time the country is among the worst in a similar comparative analysis including postcommunist Central and Eastern European countries (21, 22), the overall performance of systems in BRICS countries should be assessed as poor. This kind of conclusion will probably be true even despite the fact that the previous analysis mentioned was based on a partly different catalog of markers for health system outcomes. Jakovjlevic has also noticed significant vulnerabilities in BRICS health systems, when compared to G7 health system countries (41). This problem is being raised by decision makers in declarations published in subsequent BRICS summits. During the summit held in Sanya, China in 2011 health objectives has been highlighted, with particular focus on ensuring universal access to health services and the issue of growing costs of communicable and noncommunicable diseases (42). Also, the summit in Ufa in 2015 outlined the priorities for health systems, with HIV/AIDS, tuberculosis, malaria, neglected tropical diseases, polio, and measles to be the main issues to be addressed (42).

## Limitations of the Study and Applied Methodology

Our study is one of the first to address comparison and assessment of health systems in BRICS countries, and the methodology we use is characterized by a number of advantages. Firstly, it provides a comparative perspective for health system outcomes in investigated countries, based on data that may be retrieved from publicly available databases, making it relatively easy to be verified or repeated. The formula we propose gives the opportunity to compare the systems in individual countries in an illustrative and informative way, while giving the opportunity to also capture time trends. At the same time, the formula we propose for normalizing and aggregating the data is relatively simple and flexible enough to be subject to modifications where necessary, although, obviously the study does not provide an unequivocal answer to a question, which indicators are compulsory or the best ones to assess health system outcomes in a given country or a group of countries.

There are, however, several limitations of the applied methodology. Firstly, since we are basing on secondary data sources, no verification of their correctness and accuracy is possible. The problem might be solved by a multisource verification of the data. In some cases, however, there is a general lack of them, where at the same time such additional verification would complicate the analytical procedure, limiting its basic advantages. Additionally, we had to face shortages of data in some cases, which on the one hand resulted in limited catalog of indicators to be finally included in the synthetic measure, and secondly appears in incomplete time series in some cases. Although this does not frequently appear in the case of indicators we decided to use and should not significantly affect the accuracy of the result of our analysis, undoubtedly the replacement of actual data with statistical extrapolation does not allow a fully accurate reflection of reality and may result in deviations in some cases. Finally, the aggregation of health system outcome indicators provides a clear and easy-to-read comparison of how examined countries perform, but it does not allow to catch direct influences of individual determinants on particular outcomes. It does not also provide a background for the assessment of the strength of individual components' impact on the final result of a given country. Objectivization of weights to be attributed to individual components should be subject to further development of the proposed methodology to eliminate this limitation.

Apart from limitations related to the construction of the synthetic measure, the methods we used for testing correlation between variables cannot provide an answer regarding what the direction of relationship is or the dependence between individual variables and health system outcomes. Additionally, our study does not let to draw the objective picture of health care systems in BRICS countries, which might help to identify good practices applied in their governance or interventions implemented to modify the health status of the population. Being only of comparative nature, it does not provide an evaluation of health systems in BRICS countries in objective terms, i.e., compared to countries that might act as benchmarks. Both of these issues should be subject to future studies.

## Conclusions

1. Among the BRICS group countries, Russia, Brazil, and China receive the best score in terms of health system outcomes, as reflected with aggregated measure consisting of indicators related to infant and maternal mortality rates, life expectancy, tuberculosis detection rate, and immunization, with relatively small differences between these countries starting from 2010. South Africa and India are getting a noticeably worse result. Time trends reveal a stable situation in Russia and Brazil and regular, systematic improvement in China, which gets the best result starting from 2010, although with no further improvement when compared to remaining two countries. Also, India managed to improve its health system outcomes, with a regular rising trend starting from 2003. In South Africa, in turn, a strong opposite trend has been observed throughout the entire period 2000–2017.
2. Health system outcomes in BRICS countries as presented with the synthetic measure correlate in a statistically significant scale with general health expenditures per capita—both absolute and calculated in purchase power parity, government's health expenditure, GDP per capita and the share of industry in GDP. These correlations however remain weak, being just moderate in the case of government's health expenditure and GDP per capita value. In the case of individual measure components, tuberculosis detection rate tends to show the strongest correlation with macroeconomic indicators. A strong negative correlation between infant mortality and health expenditures, especially government's, is also noteworthy.
3. Our study does not provide an answer for what the reasons of weak correlations are between macroeconomic context and the health system outcomes in BRICS countries. Existing literature suggests that pooling of funds may play a particular role in determining their result, along with some additional factors like human resource deficiencies. Also, intensifying activities to reduce unhealthy behaviors in BRICS countries should be recommended to make further and more effective improvements, whereas in South Africa intensified intervention on the side of health system to tackle HIV/AIDS epidemics is necessary, as this appears to be the main factor determining this country's deterioration in terms of health system outcomes in the last two decades. Nonetheless, further studies are needed to find out to what extent and how exactly these factors affect health system outcomes in BRICS countries. In addition, it is recommended to undertake further research to assess health system outcomes in these countries in relation to the external benchmark.

## Data Availability Statement

Publicly available datasets were analyzed in this study. This data can be found here: https://databank.worldbank.org/source/world-development-indicators.

## Author Contributions

PR conceived the study, performed all analyses, and prepared final version of the paper. AP contributed to introduction and discussion sections in the draft version of the paper. KB collected the data and contributed to the results section in the draft version of the paper. TH contributed to the results interpretation and conclusions in the draft version of the paper.

### Conflict of Interest

The authors declare that the research was conducted in the absence of any commercial or financial relationships that could be construed as a potential conflict of interest.

## Footnotes

^1^https://databank.worldbank.org/source/world-development-indicators (accessed September 17, 2019).
